# “D” is for Dilemma

**DOI:** 10.7759/cureus.5669

**Published:** 2019-09-16

**Authors:** Pirkash Kumar, Carolina Borz-Baba, Sina Raissi

**Affiliations:** 1 Internal Medicine, Saint Mary's Hospital, Waterbury, USA; 2 Medicine, Saint Mary's Hospital, Waterbury, USA

**Keywords:** multiple myeloma, igd multiple myeloma, diagnosis, treatment

## Abstract

Immunoglobulin D (IgD) monoclonal gammopathy is a rare subtype of multiple myeloma (MM) associated with a worse prognosis compared with other variants of MM. A 61-year-old man with no known past medical history presented with complaints of abdominal pain, nausea, and vomiting for three weeks. Physical examination revealed mild epigastric tenderness. Laboratory data demonstrated a significantly elevated creatinine with minimal proteinuria and small abnormality in the gamma fraction. Ultrasound of the kidneys described normal-sized kidneys. Serum-free light chains and immunofixation were consistent with IgD kappa monoclonal gammopathy. Kidney biopsy revealed cast nephropathy. Bone marrow biopsy was remarkable for sheets of CD 38+ plasma cells comprising approximately 80% of the marrow cells. Recognizing the atypical presentation of IgD MM is crucial to facilitate early diagnosis and management and improve the prognosis of this subtype of MM.

## Introduction

Multiple myeloma (MM) is a neoplastic condition characterized by proliferation of plasma cells in the bone marrow, which produce monoclonal immunoglobulins. The diagnosis of myeloma is suspected by the presence of distinctive clinical, biochemical, radiographic, and pathologic features. Renal biopsy performed for unexplained renal disease frequently leads to the diagnosis of cast nephropathy [1]. Immunoglobulin D (IgD) MM is a rare subtype of myeloma, comprising around 2% of all myelomas [2]. This case discusses an atypical presentation of kappa light chains IgD MM and reviews the recent advances attained in the treatment of the disease. 

## Case presentation

A 61-year-old man arrived at the emergency department reporting abdominal pain, persistent nausea, and vomiting for three weeks. It was localized to the upper-mid abdomen and was constant, dull, and 5-7 out of 10 in intensity and non-radiating. The pain was predominantly diurnal and aggravated with food intake but did not relieve with eructation, vomiting, bowel movements, or change in position. The pain was associated with nausea, followed by nonbilious, nonbloody vomiting unrelated to eating. He had not attempted to treat the symptoms with medications. He reported a 25-pound unintentional weight loss in the last month but denied fever, jaundice, change in the color of his urine or stool, chest pain, cough, shortness of breath, diarrhea, constipation, urinary symptoms, or fatigue.

Abdominal pain associated with nausea and vomiting is a common presenting symptom for patients seeking evaluation in the emergency department. A multitude of disorders affecting various organ systems such as the gastrointestinal tract, cardiovascular, endocrine, metabolic, CNS, renal, hematological, malignant and nonmalignant processes may be associated with such symptoms. A comprehensive history and physical examination need to be conducted to identify potential etiologies to the underlying processes. 

The patient’s medical history was positive for atraumatic and intermittent back pain for the past six months. Lower back pain that persisted mostly during day-time worsened with a change in position. Family history was pertinent for high blood pressure and diabetes in both mother and brother. The patient denied taking any medications, herbal, or nutritional supplements. He denied smoking, alcohol intake, or drug use. He had not been sexually active for many years and did not have a history of sexually transmitted infection. The patient was originally from Latin America, but had not traveled outside the US in the past several years and had not seen a physician for the last 20 years. He lived alone and worked as a laborer. On physical examination, the patient appeared to be in no pain. The temperature was 97.1 °F, heart rate 81 beats per minute, blood pressure 140/88 mmHg, respiratory rate 20 breaths per minute, and oxygen saturation 100% at room air. Oral mucosa was dry. Sclera was anicteric. No conjunctival pallor was noted. The abdominal examination demonstrated normal bowel sounds, mild diffuse tenderness to deep palpation, which was most prominent over the epigastric region. There was no rigidity, guarding, or organomegaly. Murphy sign was negative. No abdominal bruits were noted. CVA tenderness was not elicited. Cardiac, respiratory, skin, musculoskeletal, and neurologic examinations were unremarkable.

Initial laboratory investigations revealed white cell count 8.5 k/uL (4.0-10.5), hemoglobin 12.1 g/dL (13.5-18.0), platelet count 203 k/uL (150-450), glucose 161 mg/dl (70-105), creatinine 10.8 mg/dl (0.7-1.3), blood urea nitrogen 65 mg/dl (7-25), bicarbonate 30 mg/dl (21-32), calcium 9.2 mg/dl (8.6-10.3), lipase 31 U/L (11-82), albumin 3.8 g/dl (3.5-5.0), globulin 3.0 g/dl, and total protein 6.8 g/dl (6.0-8.3). Hemoglobin A1c was 8.35. Urinalysis demonstrated trace leukocyte esterase, protein 30 mg/dl, and trace blood on dipstick. Microscopic analysis revealed no white blood cells, red blood cells, casts, crystals or bacteria.

The most remarkable finding on laboratory studies was the significantly reduced glomerular filtration rate. Given the history of nausea and vomiting over the past 2-3 weeks and the presence of elevated serum bicarbonate, it is likely that volume depletion and prerenal azotemia contributed to his kidney failure. On the other hand, the absence of hypotension or significant tachycardia made it unlikely that hypovolemia was the sole cause of his renal failure. The presence of mild proteinuria could be consistent with intrinsic renal disease. Elevated blood glucose and HbA1c suggested the possibility of diabetic nephropathy, which is the most common cause of end-stage renal disease, but it is highly unlikely that unrecognized diabetic nephropathy was the primary cause of renal failure in this case. 

**Figure 1:**
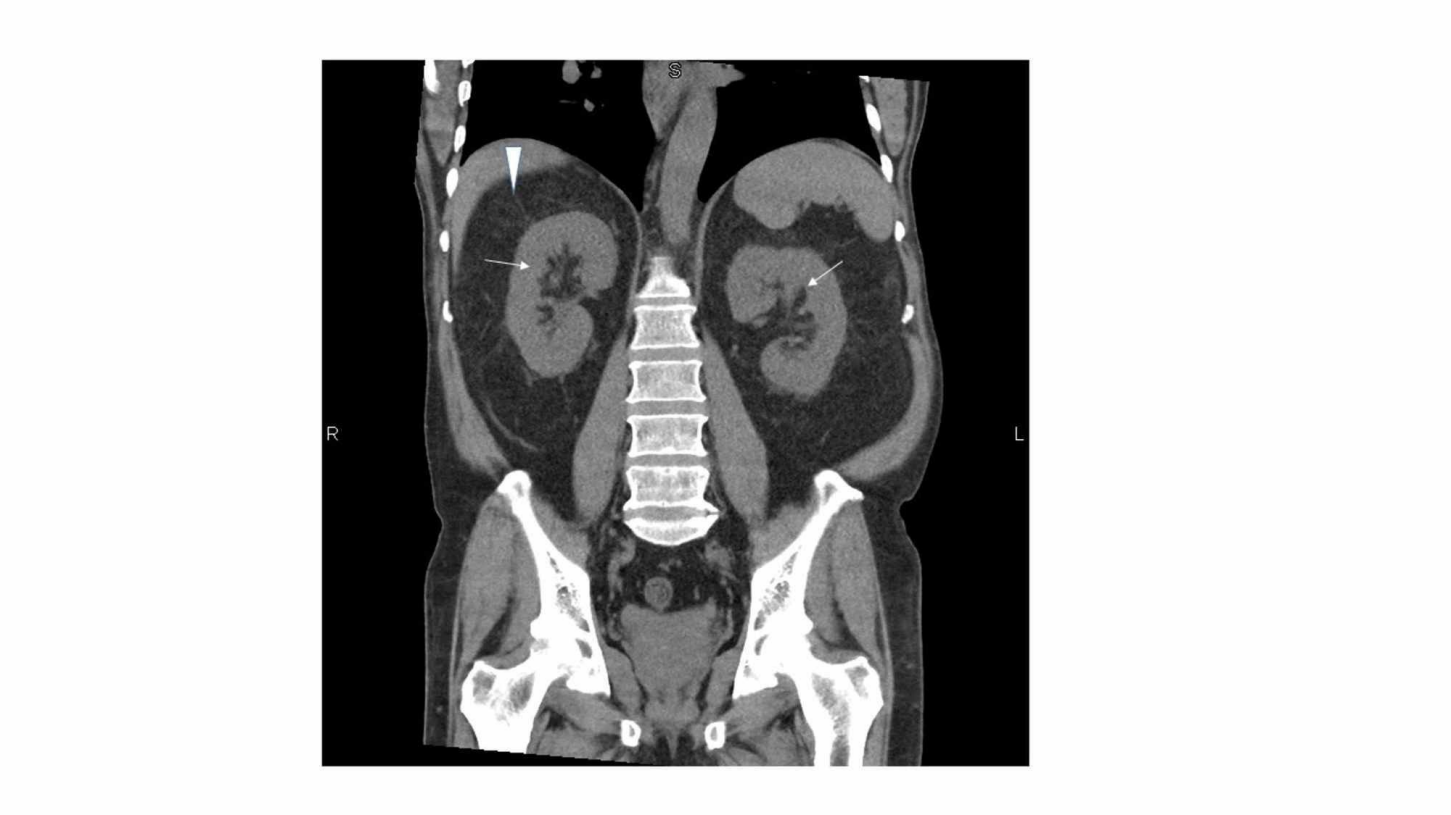
CT abdomen CT, computed tomography

Abdominal ultrasonography showed normal-sized kidneys with normal cortical thickness, no hydronephrosis or evidence of urolithiasis, and no masses. The right kidney measured 11.7 cm longitudinally and the left kidney measured 12.3 cm longitudinally. Liver, spleen, gallbladder, and bile ducts were normal in size and appearance. A non-contrast computed tomography (CT) scan (Figure 1) of the abdomen obtained in the emergency room confirmed normal size kidneys (arrows) and nonspecific bilateral perinephric stranding (arrowhead) and descending and sigmoid colon diverticulosis.

The patient initially received 2 L of intravenous crystalloid that was stopped when he developed dyspnea and jugular vein distension. His urine output over 12 hours was 1 liter. His vital signs remained stable, but the physical examination was consistent with a hypervolemic state. Creatinine levels decreased from 10.8 to 10.1 mg/dL.

The minimal improvement in kidney function with volume expansion excluded prerenal azotemia as the main cause of kidney failure. The imaging studies effectively ruled out the possibility of obstructive uropathy. The most striking feature of his imaging studies was the absence of gross evidence of chronic kidney damage such as decreased kidneys, increased echogenicity, decrease in cortical thickness, or cyst formation. A number of chronic kidney diseases, such as diabetic nephropathy, amyloidosis, HIV nephropathy, and autosomal dominant polycystic kidney disease, can be associated with preserved, or even enlarged kidney size despite advanced kidney failure. Alternatively, the imaging findings were consistent with an acute process or subacute process, resulting in a significant reduction in renal function over the span of 2 to 3 weeks before admission but unchanged kidney size. Such acute and subacute processes might include acute tubular necrosis, acute allergic interstitial nephritis, acute crystal-induced nephropathy, acute glomerulonephritis, monoclonal gammopathy of renal significance, and myeloma cast nephropathy as possible differentials. Further serological workup for HIV, hepatitis viruses, serum protein electrophoresis, quantification of urine protein excretion, urine protein electrophoresis, measurement of serum complements, and tests for ANA and antineutrophil cytoplasmic antibodies including anti-PR3 and anti-MPO antibodies were obtained.

Spot urine protein/creatinine was 1.96 g/day. Serum protein electrophoresis showed a small abnormality in gamma fraction. Serological studies for anti-PR3 and anti-MPO antibodies and ANA were negative. Serum complements were within the normal range. Hepatitis screen and HIV serology were also negative.

Despite only trace urine protein detected on the dipstick, spot urine protein to creatinine ratio indicates the presence of substantial proteinuria. This finding associated with the abnormality detected on serum protein electrophoresis was indicative of potential paraproteinemia-related kidney disease. On subsequent serum immunofixation and serum-free light chain assay, he was found to have a very elevated serum kappa free light chains 1240 mg/dl (normal range: 0.3300 to 1.94 mg/dl) and kappa/lambda ratio 816 (normal range: 0.2600 to 1.65).

**Figure 2:**
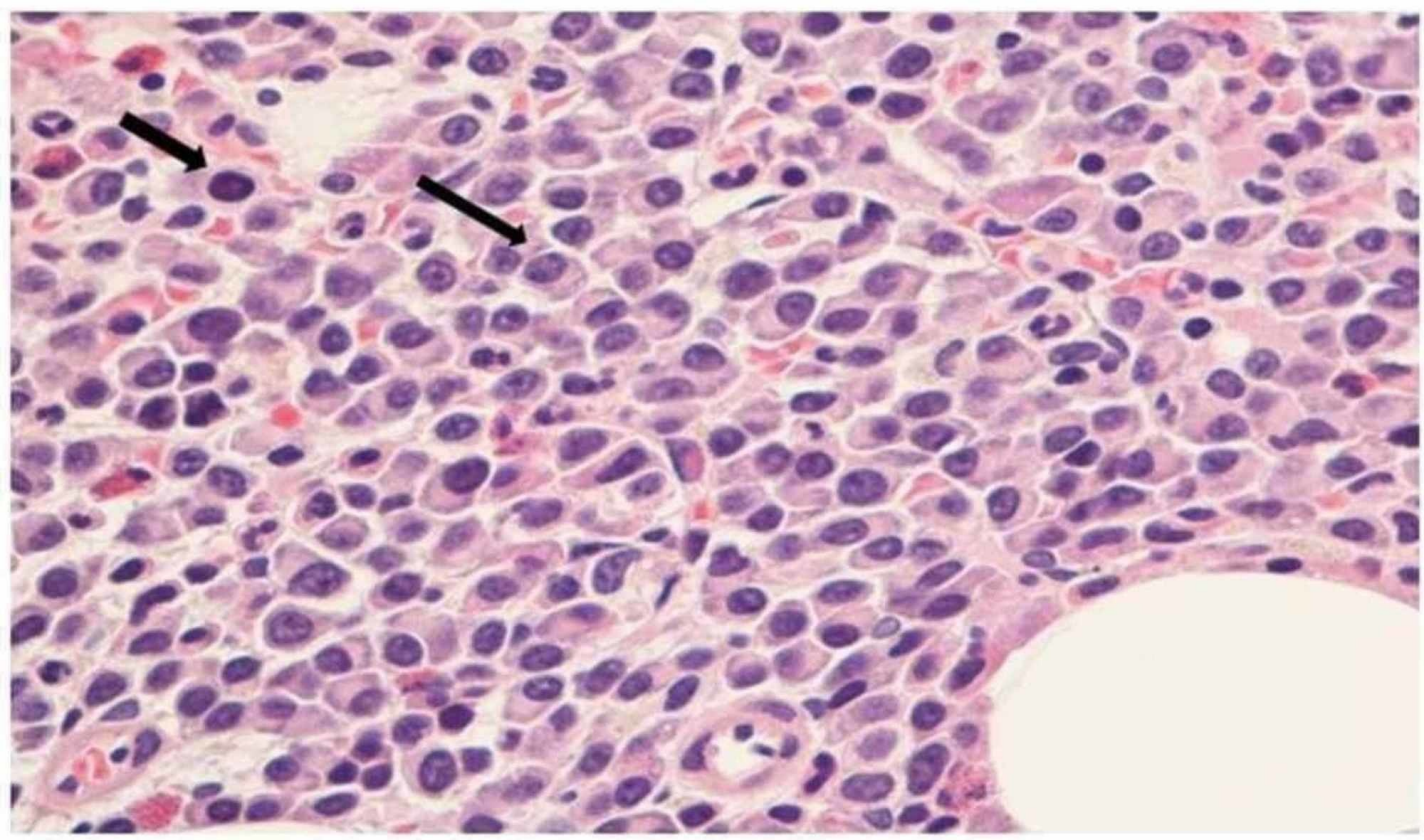
Bone marrow biopsy

Severe renal failure in the presence of very high levels of free light chains is diagnostic of monoclonal gammopathy. To accurately assess the characteristics of the monoclonal gammopathy, the patient underwent bone marrow and renal biopsies. Bone marrow biopsy (Figure 2) depicted sheets of plasma cells (arrows) comprising up to 80% of marrow cells in some areas.

**Figure 3:**
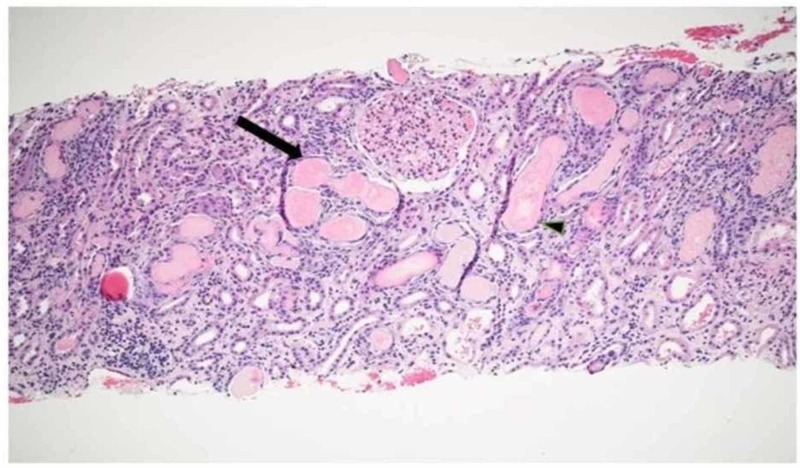
Renal biopsy

Renal biopsy (Figure 3) demonstrated tubules containing casts of glassy to slightly granular proteinaceous material (arrow). The tubular epithelium appeared attenuated and flattened in some area (arrowhead) consistent with cast nephropathy.

The diagnosis of IgD MM with myeloma cast nephropathy was confirmed. Skeletal survey was obtained, which was normal. The patient’s urine output remained stable and he did not require hemodialysis.

The patient received one cycle of cyclophosphamide 1,692 mg intravenously with subcutaneous bortezomib of 2.4 mg and dexamethasone 40 mg intravenously. Acyclovir of 200 mg daily was initiated for Varicella zoster prophylaxis. The patient was discharged after the first cycle of chemotherapy. His creatinine level has decreased to 7.3 mg/dl by the day of discharge from 10.5 mg/dl on admission. He has since completed three cycles of chemotherapy and his creatinine level has improved significantly to 1.5 mg/dl. 

## Discussion

MM accounts for 1% to 2% of all cancers [3]. The median age at diagnosis is 66 years [2]. Patients with MM commonly present with anemia (73%), bone pain (58%), renal failure (48%), hypercalcemia (28%) and weight loss (24%). Less common features include increased susceptibility to infections, neuropathy, fever, hepatomegaly, and splenomegaly [3]. The vast majority (97%) of patients with MM will have a monoclonal (M) protein secreted by the malignant plasma cells. The malignant plasma cells can produce immunoglobulin heavy chains plus light chains, light chains alone, or neither. Their frequencies are IgG (52%), IgA (21%), kappa or lambda light chain only (16%), IgD (2%), X (2%), IgM (0.5%), and negative (6.5%) [2]. The vast majority of paraproteins are kappa light chain isotype (2:1 ratio) with the exception of IgD myeloma and myeloma associated with amyloidosis, where there is a lambda light chain predominance [4].

Appropriate hematologic testing is crucial and it conventionally includes serum protein electrophoresis (SPEP), urine electrophoresis (UPEP), immunofixation, and serum free light chain (FLC) assays. SPEP permits detection and quantification of M-component but is not the most sensitive test. Serum immunofixation confirms the presence of the M-protein and determines its type. Serum FLC assay is very sensitive and specific in determining a monoclonal gammopathy and provides accurate quantification of kappa and lambda light immunoglobulin chains that are unbound to heavy chains in the serum [1].

Bone marrow aspirate and biopsy are essential to characterize the severity of disease and establish therapeutic strategies.

In patients with renal insufficiency, kidney biopsy with immunofluorescence and electron microscopy is not only diagnostic but also excludes other causes of acute renal failure and can differentiate between light chain disease and other forms of myeloma-related nephropathy. The two principal categories of renal disease associated with monoclonal gammopathies are distinguished based on the burden of the underlying plasma cells [5]. High tumor mass proliferation due to the production of large amounts of monoclonal Ig represents the group of renal disease-related monoclonal gammopathy that requires immediate therapy [5]. Myeloma kidney with cast nephropathy, like in our patient case, is the classic presentation of the group of diseases associated with monoclonal gammopathy. The other category is represented by low-grade lymphoproliferative disorders that are recently reconsidered under the name of monoclonal gammopathy of renal significance (MGRS) which includes patients who do not fulfill the criteria for multiple myeloma/ B-cell proliferation [5].

IgD myeloma is an uncommon subtype, which was first reported by Rowe and Fahey [6]. It has been recognized in relatively younger patients, with a median age at diagnosis of 52-60 years [7]. Clinical features of IgD myeloma are similar to those of IgG and IgA myeloma and include bone pain, weakness, anemia, renal failure, fatigue, and weight loss [4]. Skeletal involvement was also noted to be much common in IgD myeloma [4,8]. Typically there is a small or absent M spike in protein electrophoresis, which could be very challenging to practitioners who expect the typical “M spike” or paraprotein on serum protein electrophoresis. Elevated creatinine levels >2 mg/dL are seen in 33% to 54% of cases with IgD myeloma [4,9]. Primary kidney involvement and severe renal insufficiency are seen in only 15% to 20% of cases [1]. Light chain cast nephropathy is the most common pathophysiologic mechanism leading to renal failure in this subtype of MM. The filtered monoclonal light chains form intra-tubular casts that obstruct the urine flow, incite foreign body reaction, and can cause tubular fibrosis. In addition, light chains can also cause direct toxicity to proximal tubular cells and intracellular crystal formation. Lambda light chain is more common in IgD myeloma [4].

Our patient presented with nonspecific complaints including abdominal pain, nausea, and vomiting and was found to have significant renal failure. He did not have any typical features, suggestive of myeloma such as anemia, hypercalcemia, significantly elevated serum proteins, or large proteinuria. He also presented with primarily kidney involvement and severe renal insufficiency, which are infrequently seen in IgD myeloma [1]. To add to the unusual, our patient’s myeloma clone produced kappa light chains clone and not lambda, which is more prevalent. The subtype of IgD carries an important prognostic value, as the presence of kappa variant along with a WBC count < 7.0 10 x 9/L at diagnosis are associated with prolonged survival [9].

Prior to the availability of novel therapeutic agents and the use of autologous stem cell transplantation (ASCT), the survival rate was less than two years [4]. Therapeutic options for IgD myeloma have recently made significant advances which include novel agents (thalidomide, bortezomib, and lenalidomide) and ASCT. Recent studies comparing outcomes following chemotherapy alone vs ASCT reported a significant benefit in survival when patients are treated with high-dose therapy followed by ASCT [10-13]. With the use of novel agents (thalidomide, bortezomib, and lenalidomide) and ASCT, survival in patients with IgDMM has improved although it remains inferior to that of IgG, IgA, and light chain [4,10,14-15].

## Conclusions

The variety of renal involvement in MM is ample. Ig D myeloma kidney is a rare entity which may be difficult to diagnose due to potentially atypical symptoms and unrevealing protein electrophoresis. Patients with small or absent M spike on protein electrophoresis, unexplained renal failure, or bone pain should be further investigated for IgD myeloma. Enhanced cognizance of this disease may improve early detection and treatment and subsequently translate into better patient outcome.
